# Twenty Years after De Ley and Blaxter—How Far Did We Progress in Understanding the Phylogeny of the Phylum Nematoda?

**DOI:** 10.3390/ani11123479

**Published:** 2021-12-07

**Authors:** Mohammed Ahmed, Oleksandr Holovachov

**Affiliations:** Department of Zoology, Swedish Museum of Natural History, 114 18 Stockholm, Sweden; mohammed.ahmed@nrm.se

**Keywords:** nematoda, molecular phylogeny, 18S rRNA, phylogenomics, classification, evolution

## Abstract

**Simple Summary:**

New technological advancements often radically change our views on the world. Sequencing of DNA and use of it to reconstruct the evolution of organisms was an advancement that revolutionised human understanding of the origin of nematodes and interrelationships within this often neglected but diverse and important group of animals. This manuscript provides a summary of current knowledge and opinions on the relationships within nematodes, indicates where further research is needed and briefly discusses some of the most common problems and errors in molecular phylogeny of the phylum Nematoda.

**Abstract:**

Molecular phylogenetics brought radical changes to our understanding of nematode evolution, resulting in substantial modifications to nematode classification implemented by De Ley and Blaxter and widely accepted now. Numerous phylogenetic studies were subsequently published that both improved and challenged this classification. Here we present a summary of these changes. We created cladograms that summarise phylogenetic relationships within Nematoda using phylum-wide to superfamily-wide molecular phylogenies published in since 2005, and supplemented with the phylogenetic analyses for Enoplia and Chromadoria with the aim of clarifying the position of several taxa. The results show which parts of the Nematode tree are well resolved and understood, and which parts require more research, either by adding taxa that have not been included yet (increasing taxon coverage), or by changing the phylogenetic approach (improving data quality, using different types of data or different methods of analysis). The currently used classification of the phylum Nematoda in many cases does not reflect the phylogeny and in itself requires numerous improvements and rearrangements.

## 1. Introduction

The rise in molecular phylogenetics at the end of the 20th century revolutionised our understanding of nematode evolution [1] and resulted in many radical but logical changes in the classification implemented by De Ley and Blaxter [2,3]. Accepted by many during past two decades, this classification still included many paraphyletic and polyphyletic groups, and the ranking of many taxa was not strictly equivalent from the phylogenetic or chronological points of view. Since then, numerous phylogenetic and phylogenomic analyses have been published (cited below in the text), both supporting and rejecting various parts of the “De Ley & Blaxter’s” classification, with some changes formally introduced [4] and many remaining not formalised. Hodda [5] provided a revised classification of nematodes, introduced a rank of superorder and inflated status of many taxa to account for the uneven ranking of clades comparing to De Ley and Blaxter [2,3]. However, his classification was not accepted by nematode taxonomists. A new version is on the way [6], which we hope will be considered by colleagues more seriously and thoroughly.

Classifications are meant to be based on phylogeny, but they never truly reflect the relationships of organisms. Classifications are opinions of researchers; they are simplified systems that may have to include paraphyletic taxa, may give similar rank to clades of different age or level of branching in the cladogram. Thus, classifications that are in use can never be “converted” back into phylogenetic tree, and with so many existing phylogenetic analyses covering every level of nematode evolution, from the entire phylum to individual species, the need for “unified” nematode phylogeny and “unified” classification based on it remains high. This manuscript provides a simplified overview of a current consensus phylogeny of the phylum Nematoda up to a family-group level.

## 2. Materials and Methods

Cladograms depicting summarised relationships within Nematoda are derived from phylum-wide to superfamily-wide molecular phylogenies published since 2005, and supplemented with two phylogenetic analyses provided in the supplementary files. Para- and polyphyletic lineages are depicted as filled curvilinear triangles and trapezoids, respectively. Where there was a difference in topology or support between competing studies, the chosen topology is discussed in the appropriate place in the text of the paper, with reference to respective publication. For current phylogenetic analyses, sequences were generated as previously described [7]. Previously published alignments from [8,9] were used as templates for alignment and annotation of the recently published and newly generated sequences. Secondary structure annotation was manually added to all non-annotated sequences using the 4SALE [10], all sequences were manually aligned to maximise apparent positional homology of nucleotides and V1 and V9 hypervariable domains were trimmed off. Phylogenetic trees were built using PHASE 3 [11] with the REV GAMMA nucleotide substitution model for non-paired sites and RNA16A paired-site substitution model for the paired sites of the rRNA. 

## 3. Results

### 3.1. Basal Dichotomy

**Figure 1:**
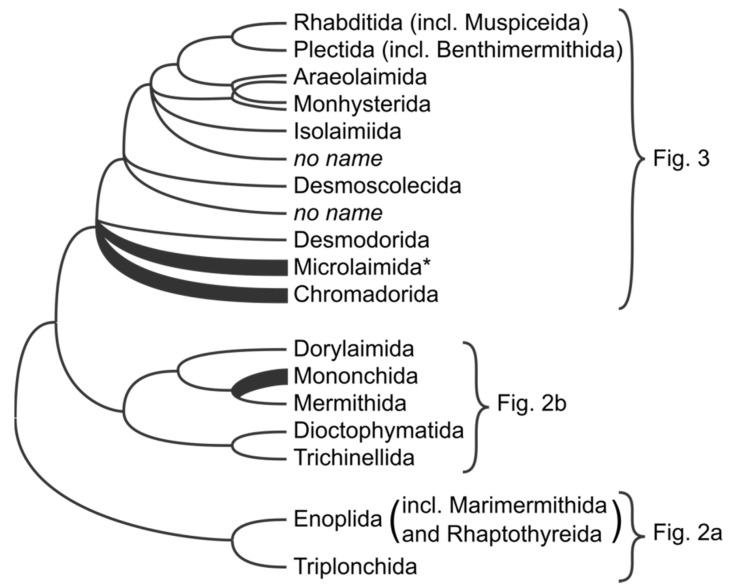
Simplified overview of phylogenetic relationships between different orders of the phylum Nematoda, based on the combination of phylogenomic and 18S rRNA-based analyses. Taxa marked with “*” were not included in the classification of De Ley and Blaxter [2,3] but have been introduced or used in subsequent publications. “*no name*” refers to distinct well-supported lineages that were formally classified in one of the existing orders but phylogenetically do not belong to those; based on the current topology they should be given a status of new orders.

The ideas of what could have been the first split in the Nematode tree have already been discussed in detail [2,12]. The basal divergence with Enoplia being sister to a clade Dorylaimia and Chromadoria (Figure 1) was firmly supported in the latest phylogenetic studies based on single [13] and multiple markers [14,15], contrary to earlier studies. Thus, recent phylogenies reject the division of Nematoda into two classes, Enoplea (including subclasses Enoplia and Dorylaimia) and Chromadorea (with single subclass Chromadoria), as proposed by De Ley and Blaxter [2,3]. 

### 3.2. Enoplia

**Figure 2:**
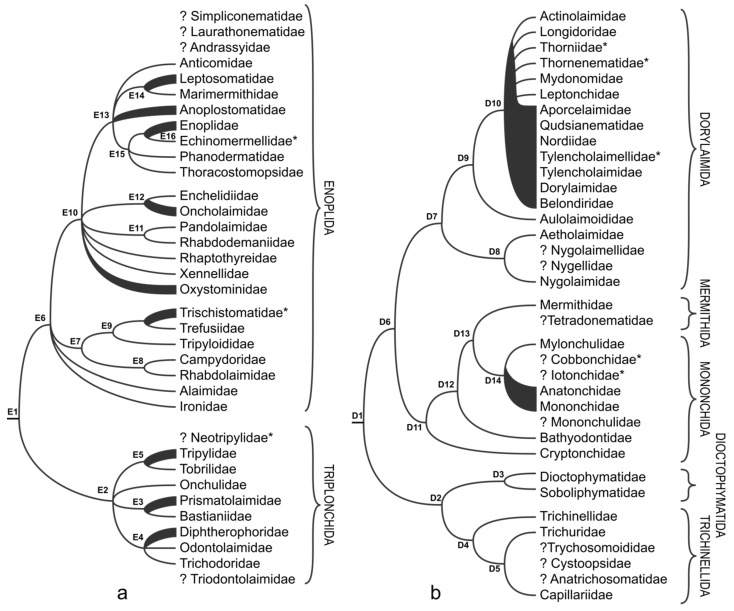
Overview of phylogenetic relationships within: (**a**) Enoplia; (**b**) Dorylaimia. Taxa marked with “*”were not included in the classification of De Ley and Blaxter [2,3]; “?” indicates taxa not included in molecular phylogenies.

The division of Enoplia into two clades (E1 in Figure 2a and Figure S1), roughly equivalent to the orders Enoplida and Triplonchida (with subsequent changes discussed below), is well supported in the majority of rRNA-based phylogenetic analyses [12,13,16,17,18]. However, both existing phylogenomic analyses that included single available transcriptome from the member of the order Triplonchida (=*Tobrilus*) do not resolve such division [14,15]. In both cases, Enoplida is paraphyletic [14,15], which can be explained by the limited taxon sampling in Triplonchida and low data completeness for some Enoplida included in both studies, especially for *Bathylaimus*, which is placed as a sister taxon to *Tobrilus*/Triplonchida.

**Figure 3:**
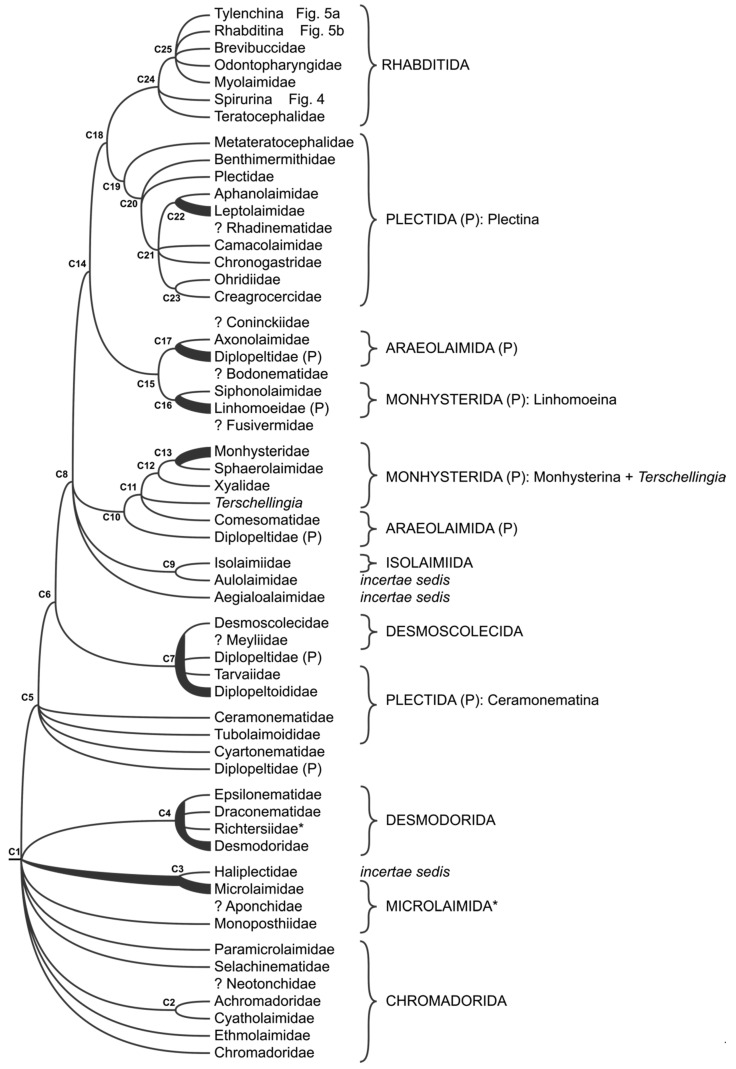
Overview of phylogenetic relationships within Chromadoria. Taxa marked with “*”were not included in the classification of De Ley and Blaxter [2,3]; (P) marks polyphyletic taxa; “?” indicates taxa not included in molecular phylogenies.

**Figure 4:**
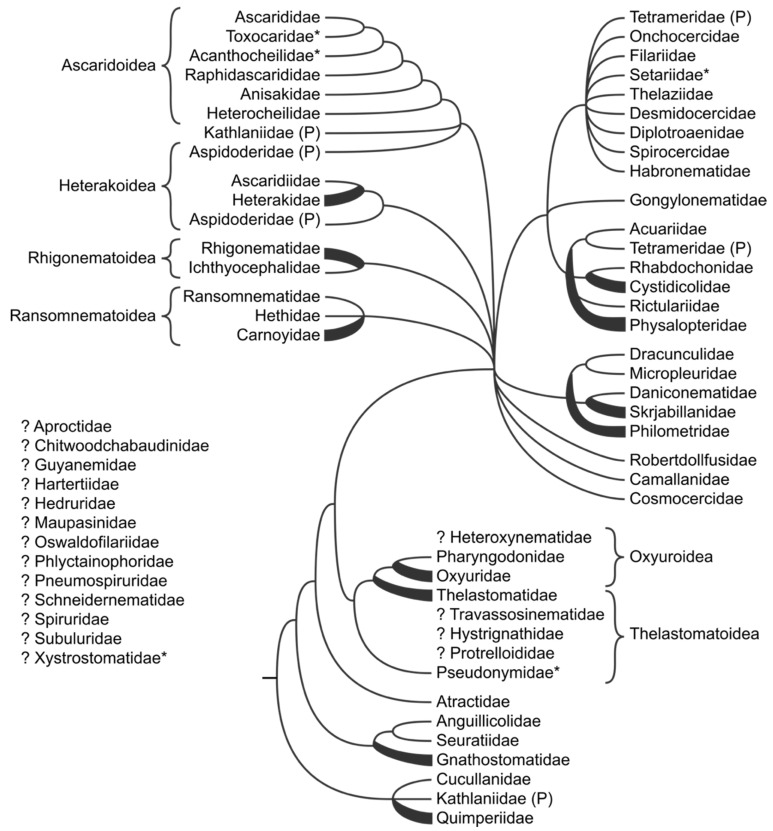
Overview of phylogenetic relationships within Spirurina. Families marked with “*”were not included in the classification of De Ley and Blaxter [2,3]; (P) marks polyphyletic taxa; “?” indicates taxa not included in molecular phylogenies.

Phylogenetic relationships within the order Triplonchida (Figure 2a) are not fully resolved based on the 18S rRNA data and do not fully agree with the classification proposed by De Ley and Blaxter [3]. The basal polytomy splits into four clades (E2 in Figure 2a): (1) Onchulidae so far represented by a single sequenced species; (2) clade consisting of Bastianiidae (placed in the order Plectida [3]) and Prismatolaimidae, weakly supported in some analyses (E3 in Figure 2a); (3) clade with Tobrilidae being nested within the paraphyletic Tripylidae (E5 in Figure 2a); (4) clade including Trichodoridae, Odontolaimidae (placed in the order Plectida [3]) and polyphyletic Diphtherophoridae (E4 in Figure 2a) [12,19,20,21,22] (Figure S1). The monophyly of clade uniting Bastianiidae and Prismatolaimidae (E3 in Figure 2a) receives support from morphological data—both share the same unique shape of supplements and the amphid. The presence of protrusible odontostyle and male supplementary organs distributed along the ventral side of anterior body region are the two characters that support the monophyly of the clade Diphtherophoridae and Odontolaimidae and Trichodoridae (E4 in Figure 2a). The interrelationships between Tobrilidae and Tripylidae (E5 in Figure 2a) remain unresolved both from the molecular [19,20] and morphological points of view, in the latter case due to the existence of recently described *Neotripyla* (family Neotripylidae), which possesses features intermediate between two families [23]. 

Phylogenetic relationships within Enoplida (Figure 2a), as inferred based on 18S rRNA, are poorly resolved in nearly all studies [13,16,17,18,21,24,25] except for [26] who used Bayesian Inference and a very limited dataset. The cladogram depicted on the Figure 2a is in part based on our own analysis presented in the Supplementary Materials Figure S1. The basal polytomy (E6 in Figure 2a) splits into four clades. In published analyses Ironidae is clustered either with Rhaptothyreidae [27] (placed in a separate order in early publications [2,3]) or with Alaimidae [17,24], although more often Alaimidae is placed on its own in the basal Enoplida polytomy. Here, both Ironidae and Alaimidae are treated separately.

The topology of the next clade (E7 in Figure 2a) is well defined and it splits into two lineages. The first includes the families Rhabdolaimidae and Campydoridae (E8 in Figure 2a) that always form a well-supported monophyletic clade, also confirmed by morphology-based studies [28]. The second consists of Trefusiidae and Trischistomatidae and Tripyloididae (E9 in Figure 2a) that are also usually grouped in a well-supported monophyletic clade [9,17,24], although the data quality may affect the support for the monophyly of the family Trefusiidae [25]. Within this clade, monophyletic Trefusiidae is nested within paraphyletic Trischistomatidae [19,20] (Figure S1), and the placement of Trischistomatidae in Enoplida, instead of Triplonchida (previously placed in the family Tripylidae [2,3]) is supported by the morphological evidence [29]. The last clade leads to another polytomy that includes six lineages (E10 in Figure 2a).

Of these six lineages, the family Oxystominidae is represented by three separate but well-supported clades that do not group together in a monophyletic lineage in any of the analyses. Only one phylogeny groups Oxystominidae in a monophyletic clade with Oncholaimidae and Enchelidiidae [24], albeit still maintaining three separate lineages of Oxystominidae. The same polytomy includes Rhaptothyreidae, whose position in our analysis is different from that previously published [27]; newly sequenced Xennellidae (Figure S1); a clade, which until now was represented by a single species from the family Rhabdodemaniidae [18], but in our phylogeny includes several sequences from Pandolaimidae and Rhabdodemaniidae (E11 in Figure 2a). Another lineage is composed of the families Oncholaimidae and Enchelidiidae (E12 in Figure 2a), which are always grouped in a well-supported monophyletic clade, but monophyletic Enchelidiidae are deeply nested within paraphyletic Oncholaimidae, as evidenced by both single-marker (18S rRNA) and multigene analyses [14,15,17,18,24]. 

The last, largest clade is typically well supported in various analyses and splits into polytomy of its own (E13 in Figure 2a) [9,18,24], with paraphyletic Anoplostomatidae, monophyletic Anticomidae, a clade leading to paraphyletic Leptosomatidae with animal parasitic Marimermithidae deeply nested within it (E14 in Figure 2a) and a clade splitting into a trichotomy (E15 in Figure 2a) with monophyletic Thoracostomopsidae, monophyletic Phanodermatidae and paraphyletic Enoplidae, which, like Leptosomatidae above, includes a deeply nested animal parasitic Echinomermellidae (E16 in Figure 2a).

### 3.3. Dorylaimia

The pattern of early branching of Dorylaimia is conserved and stable in most (if not all) 18S rRNA-based analyses (Figure 2b), with the trichotomy at the root leading to well-supported Dorylaimida, Trichinellida and Dioctophymatida and Mononchida and Mermithida [12,13]. Phylogenomic studies, however, support a slightly different topology, with Trichinellida and Dioctophymatida forming a sister to the rest of the Dorylaimia lineage (D1 in Figure 2b), and Dorylaimida being placed in a monophyletic clade with Mononchida and Mermithida [14,15] The latter topology is adopted here (Figure 2b). Thus, one of the clades stemming from the first dichotomy (D1 in Figure 2b) leads to a well-resolved split into branches equivalent to orders Dioctophymatida (D3 in Figure 2b), represented by two families with just one sequenced member each, and Trichinellida (D4 in Figure 2b). The latter order is represented by three out of six families in molecular phylogenies. The branching pattern is as follows: the Trichinellidae is a sister clade (D4 in Figure 2b) to a lineage splitting into Trichuridae and Capillariidae (D5 in Figure 2b) [13]. The remaining three families belonging to Trichinellida [2,3], namely Trichosomoididae, Cystoopsidae and Anatrichosomatidae, have no molecular data available.

The other clade derived from the basal dichotomy of Dorylaimia splits into Dorylaimida and Mononchida and Mermithida (D6 in Figure 2b) in phylogenomic studies [14,15], as described above (Figure 2b). Dorylaimida further splits into two clades equivalent to Nygolaimina and Dorylaimina (D7 in Figure 2b), in full agreement with morphology-based classification [2,3] and molecular phylogenies [12,13,21,30]. After that, the topologies of currently available molecular phylogenies provide very little information. The suborder Nygolaimina in 18S rRNA-based molecular phylogenies is represented by members from two families, Nygolaimidae and Aetholaimidae (D8 in Figure 2b) that form a clade [31]. The other two currently recognised families, Nygolaimellidae and Nygellidae, have not yet been sequenced. Within Dorylaimina, all families have at least one species sequenced and included in either 18S or 28S rRNA-based analyses. However, in 18S rRNA-based phylogenies, only the families Longidoridae and Actinolaimidae are monophyletic within the para-/polyphyletic assemblage of other families (D10 in Figure 2b). Sequenced members of all other families are not resolved into monophyletic clades, mainly because of low variability of chosen phylogenetic marker and morphology-based classification being based on characters with high plasticity and homoplasy [30,31,32,33,34]. Similarly, partial 28S (D2D3) rRNA-based phylogeny of Dorylaimida does not resolve the relationships within Dorylaimina either. It must be noted, however, that some molecular phylogenies that do not include all available sequences may support the monophyly of several families whose taxonomic composition differs from that in De Ley and Blaxter, or which have not been recognised by them, such as Leptonchidae *sensu stricto* [35], and such analyses must be treated with caution. Furthermore, the families Aulolaimoididae, positioned at the base of Dorylaimina (D9 in Figure 2b), and Mydonomidae and Thorniidae, deeply nested within paraphyletic assemblage (D10 in Figure 2b), are so far represented by single sequences or multiple sequences from the same genus, which prevents us from investigating their monophyly [13,36,37].

The topology of the clade uniting Mononchida and Mermithida (D11 in Figure 2b) is resolved much better in all available molecular phylogenies, with the consistent branching pattern (Figure 2b). The clade representing the family Cryptonchidae is a sister to the remaining taxa (D11 in Figure 2b), followed by Bathyodontidae (D12 in Figure 2b) [13]. The next node produces two clades, one of which is represented by single sequence of a mononchid species *Granonchulus*, which has not been re-sequenced yet; its placement strongly contradicts morphology-based studies and should not be accepted until further molecular data are available. Subsequent dichotomy (D13 in Figure 2b) divides Mermithida and Mononchina. Within Mermithida, all currently available sequences belong to the family Mermithidae, resolved as monophyletic in rRNA-based phylogenies, while the other family, Tetradonematidae, from the same order, is missing from published studies [13]. The phylogeny of Mononchina is well represented, with members from all three families listed in De Ley and Blaxter [2,3] being included in phylogenetic analyses. Only Mylonchulidae is unequivocally resolved as monophyletic, while Mononchidae and Anatonchidae are usually paraphyletic or polyphyletic (D14 in Figure 2b), depending on the particularities of the analysis [38,39]. The families Iotonchidae and Cobbonchidae, not included in De Ley and Blaxter [2,3] but accepted in subsequent classifications [40], have not been included in molecular phylogenetic analyses yet.

### 3.4. Chromadoria

Phylogenetic relationships within the subclass Chromadoria remain poorly understood (Figure 3 and Figure S2). The basal polytomy (C1 in Figure 3) includes paraphyletic order Chromadorida, paraphyletic order Microlaimida, monophyletic order Desmodorida (C4 in Figure 3) and a branch leading to the rest of Chromadoria (C5 in Figure 3) [7,13,30]. None of the analyses since [41] were able to resolve Chromadorida as monophyletic [4,7,13]. Within it, only families Achromadoridae and Cyatholaimidae usually form a monophyletic clade (C2 in Figure 3), while other analysed families are part of a basal polytomy. Similarly, even the analysis of an expanded and refined dataset representing the order Microlaimida (proposed after De Ley and Blaxter [2,3]) does not support its monophyly [4] (Supplementary Materials Figure S2). Placement of the order Desmodorida is different depending on the analysis or dataset: it can be part of a well-supported dichotomy [41,42], be an ingroup within Chromadorida [15] or part of the polytomy described above [4,7]. Within the order Desmodorida, the family Desmodoridae is paraphyletic (C4 in Figure 3) and includes monophyletic Draconematidae, Epsilonematidae and Richtersiidae (Figure S2).

The subsequent (C5 in Figure 3) node also splits into polytomy that includes Diplopeltidae (only the genus *Campylaimus*), Cyartonematidae, Tubolaimoididae and Ceramonematidae—families originally classified in different orders, with only Tubolaimoididae and Ceramonematidae being morphologically similar [43]. This topology is based on a recent analysis that includes new sequences of *Campylaimus, Cyartonema* and *Tubolaimoides* (Supplementary Materials Figure S2). Next node (C6 in Figure 3), also largely based on recent analysis (Supplementary Materials Figure S2) forms a well-supported dichotomy that leads to a monophyletic clade (C7 in Figure 3) represented by the families Diplopeltoididae, Tarvaiidae, Diplopeltidae (only genus *Mudwigglus*) and Desmoscolecidae—Diplopeltoididae is paraphyletic, Desmoscolecidae is monophyletic and Diplopeltidae and Tarvaiidae are represented by one taxon each. The close relationships between Tarvaiidae and Desmoscolecidae were shown before [42], but the inclusion of Diplopeltoididae in the same lineage is new. 

The following node (C8 in Figure 3) is again represented by a polytomy that includes Aegialoalaimidae, Isolaimiidae and Aulolaimoididae—taxa which never had a stable position in the nematode classification. Close affinities between Aulolaimoididae and Isolaimiidae (C9 in Figure 3) were shown before [21], but the position of Aegialoalaimidae remains unclear [44]. The same node gives rise to a well-supported monophyletic clade (C10 in Figure 3) that defies classification of the orders Araeolaimida and Monhysterida ever since they were included in molecular phylogenies [7,21,41,42]. In the current dataset (Supplementary Materials Figure S2), it has the following topology and composition: the first split separates Diplopeltidae (C10 in Figure 3, represented by single genus *Neodiplopeltula*), formally classified in Araeolaimida; the next node (C11 in Figure 3) is a trichotomy that includes monophyletic Comesomatidae (Araeolaimida), genus *Terschellingia* (formally classified in Linhomoeina of Monhysterida) and a monophyletic clade (C12 in Figure 3) representing Monhysterina. Within Monhysterina, Xyalidae is a sister clade (C12 in Figure 3) to the lineage leading to paraphyletic Monhysteridae and monophyletic Sphaerolaimidae (C13 in Figure 3).

The subsequent node (C14 in Figure 3) is also a well-supported dichotomy, one of the clades is a well-supported monophyletic lineage (C15 in Figure 3) that also includes members of the order Monhysterida, suborder Linhomoeina (C16 in Figure 3) represented by paraphyletic Linhomoidae and monophyletic Siphonolaimidae, and members of the order Araeolaimida (C17 in Figure 3) represented by the remaining sequenced species of the family Diplopeltidae (paraphyletic) and monophyletic Axonolaimidae [7,13,21] (Supplementary Materials Figure S2). The last large division (C14 in Figure 3) separates monophyletic Plectida and Rhabditida. Within Plectida (C19 in Figure 3), Metateratocephalidae branch off first, Plectidae second (C20 in Figure 3) and the next node is a polytomy that includes Camacolaimidae, Chronogastridae, Ohridiidae and Creagrocercidae and Leptolaimidae and Aphanolaimidae. Of these, only Chronogastridae (not shown) and Leptolaimidae are paraphyletic [7,13,45]. The position of the family Benthimermithidae varies in different analyses from being sister to Plectida [7] to being one of the clades within Plectida (C20 in Figure 3) [46,47].

The early branching within Rhabditida starts with trichotomy of Teratocephalidae, Spirurina and the rest of Rhabditida (C24 in Figure 3). The next node is best represented by a polytomy (C25 in Figure 3), due to conflicting results from different datasets and inference methods [48]. The placement of families Brevibuccidae and Myolaimidae is best described by the word “unsettled”, while that of Odontopharyngidae here is likely due to long branch attraction (C25 in Figure 3). Monophyletic (based on 18S rDNA and nuclear protein-coding genes) Tylenchina and Rhabditina also originate from this node. Phylogenies based on mitochondrial genomes, however, propose an alternative topology with both Tylenchina and Spirurina being polyphyletic [49]—this incongruence is discussed in detail elsewhere [15]. 

### 3.5. Spirurina

Phylogenetic relationships within the suborder Spirurina remain insufficiently known, and the latest 18S rRNA-based phylogenies [13,50,51] present very confusing results that are difficult to interpret and describe, with many paraphyletic and polyphyletic families (Figure 4). In part this is due to presence of “rogue” taxa, the impact of which on “labelling” the phylogeny with taxonomic names (family names) has been discussed by Nadler et al. [52] specifically in the context of Spirurina. Genomic datasets, on the other hand, are too limited in taxon sampling to be able to define the phylogenetic relationships within the suborder Spirurina [14,15]. Analyses based on single gene markers and focusing on smaller subgroups within the suborder Spirurina can also produce contradicting results, as exemplified in [53] who used 18S rRNA and COI to infer the relationships within the infraorder Spiruromorpha. Only three superfamilies are resolved as monophyletic in existing molecular phylogenies: the Ascaridoidea [54], including its six families, as well as Rhigonematoidea and Ransomnematoidea [55,56], but only in some of the analyses. Thus, the cladogram presented on Figure 4 should be taken with great caution, since it is based almost exclusively on single 18S rRNA-based phylogeny [13] with some modifications from few other publications mentioned above. For the same reasons, it will not be discussed in detail in the present manuscript.

The phylogenetic relationships of the order Muspiceida, placed in Dorylaimia in De Ley and Blaxter [2,3], remain unclear. The 18S rRNA-based phylogeny places a single member of the family Robertdollfusiidae, *Haycocknema perplexum*, within suborder Spirurina, order Rhabditida [13]. The placement of the family Muspiceidae, also represented by single species, *Riouxgolvania kapapkamui*, within Rhabditina, order Rhabditida in 18S rRNA-based phylogeny is questionable, as the only available sequence shows signs of long branch attraction [13].

### 3.6. Tylenchina

**Figure 5:**
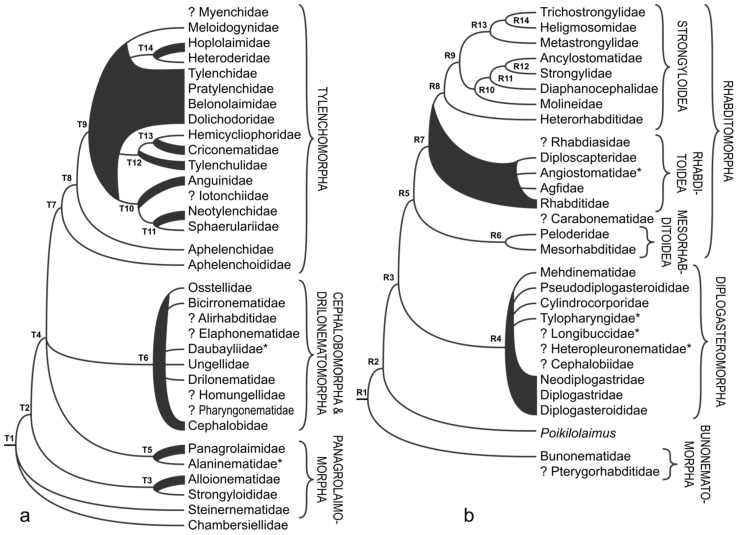
Overview of phylogenetic relationships within: (**a**) Tylenchina; (**b**) Rhabditina. Families marked with “*”were not included in the classification of De Ley and Blaxter [2,3]; “?” indicates taxa not included in molecular phylogenies.

The family Chambersiellidae is placed at the base of Tylenchomorpha (T1 in Figure 5a)—its position varies considerably depending on the data and methods used [13,48]. The relationships between the infraorders included in Tylenchina [2,3] differ depending on the type of data involved. While 18S rRNA gene data suggests a closer relationship between Tylenchomopha and Cephalobomorpha, genome-wide analysis shows that Cephalobomorpha forms a clade with Panagrolaimomopha, specifically Panagrolaimoidea instead [15], albeit with suboptimal support. This incongruence is reflected in polytomy T4 in the Figure 5a. In any case, Panagrolaimomorpha is always paraphyletic, while Tylenchomorpha and Cephalobomorpha (which includes Drilonematomorpha as an ingroup) are monophyletic in phylogenies based on 18S rRNA or nuclear protein-coding genes. 

Within Panagrolaimomorpha, the superfamily Strongyloidoidea emerged earlier in the phylogeny in two separate splits, one giving rise to Steinermatidae (T1 in Figure 5a) and the other to Strongyloididae and Alloionematidae lineage (T2 in Figure 5a). The families Alloionematidae and Strongyloididae, both of which belong to the superfamily Strongyloidea, are placed in a well-supported clade (T3 in Figure 5a); while the latter’s monophyly is supported, the former is recovered as paraphyletic [13,57]. Phylogenomic analysis places the family Panagrolaimidae in a clade with Cephalobomorpha [15]. Phylogenies based on the 18S rRNA gene as mentioned above have resulted in the placement of the family within a poorly supported clade with Aphelenchoididae [16,58], and occasionally with Strongyloididae and Alloionematidae [13,21]. The family Panagrolaimidae itself is paraphyletic (T5 in Figure 5a) in relation to the family Alaninematidae [59] (not included in [2,3]).

The relationships between the three families of the infraorder Cephalobomorpha sampled so far in molecular phylogenetic analyses (Cephalobidae, Osstellidae and Bicirronematidae) are not resolved (T6 in Figure 5a). Both Osstellidae and Bicirronematidae are represented by a single taxon in published analyses, while the largest family of this infraorder, Cephalobidae, is paraphyletic [13,60]. It also includes Daubayliidae (not included in [2,3]) [48] and Drilonematomorpha, represented by Drilonematidae and Ungellidae in published phylogenies [60,61]. The remaining families Alirhabditidae and Elaphonematidae from Cephalobomorpha and Homungellidae and Pharyngonematidae from Drilonematomorpha have not been included in molecular phylogenies.

According to 18S rRNA data, the family Aphelenchoididae has a peculiar placement with regards to the rest of Tylenchomorpha, in that while its morphological sister family Aphelenchidae occupies a position right at the base of the Tylenchomorpha clade, one that is consistent with morphology, Aphelenchoididae occupies a clade well outside Tylenchomorpha. This clade is often poorly supported and may group with members of the families Panagrolamidae, Strongyloididae and Alloionematidae [13,21,58]. Genome-wide analysis of phylogeny does, however, result in a topology consistent with morphological hypothesis [15], placing Aphelenchoididae (T7 in Figure 5a) and Aphelenchidae (T8 in Figure 5a) at the base of Tylenchomorpha (Figure 5a). The remaining taxa are united in a clade (T9 in Figure 5a), but the relationships within it are poorly resolved. Within the infraorder Tylenchomorpha, all four superfamilies, namely Tylenchoidea Criconematoidea, Sphaerulorioidea and Aphelenchoidea are not supported by 18S rRNA gene. Tylenchidae are paraphyletic or polyphyletic, depending on the analysis and classification. 

The use of 18S rRNA gene data does not resolve the relationship between the families of Sphaerularioidea, and the superfamily itself is recovered either as polyphyletic [13] or monophyletic [58]. Phylogenomics involving two of the families, namely Anguinidae and Neotylenchidae, provided strong support for the monophyly of the superfamily as well as that of the respective families, while 18S rRNA data implies both Anguinidae (T10 in Figure 5a) [58] and Neotylenchidae (T11 in Figure 5a) [13] to be paraphyletic, which, taking into consideration the limited taxon sampling in phylogenomic analysis [15], is reflected in Figure 5a. While the most recent analyses of the 18S rRNA gene provided support for the monophyly of the superfamily Criconematoidea [13], two of its three families, Tylenchulidae (T12 in Figure 5a) and Criconematidae (T13 in Figure 5a), are consistently shown to be paraphyletic [13,21,62]. Hemicyliophoridae, represented mostly by a single genus, is recovered as monophyletic and nested within paraphyletic Criconematidae. 

The remaining families of Tylenchomorpha, classified in the superfamily Tylenchoidea, are not monophyletic. The families Tylenchidae, Belonolaimidae, Pratylenchidae and Dolichodoridae often form a paraphyletic assemblage (T9 in Figure 5a) that nests within it the above-mentioned Criconematoidea and Sphaerularioidea, as well as monophyletic Meloidogynidae and a clade that unites paraphyletic Hoplolaimidae with monophyletic Heteroderidae (T14 in Figure 5a) [13,58,63]. 

The position of the superfamily Myenchoidea and family Myenchidae remain unresolved due to lack of data.

### 3.7. Rhabditina

Ribosomal RNA-based phylogenies do not clearly resolve the basal branching within Rhabditina [13]. Phylogenomic data, however, clearly supports Bunonematidae as a sister to the rest of Rhabditina [15], even though it is represented by a single species (R1 in Figure 5b). Bunonematidae in itself is monophyletic in 18S rRNA-based phylogenies, while the other family considered closely related to it based on morphological characters, Pterygorhabditidae, has not been sequenced yet. The subsequent node (R2 in Figure 5b) produces a clade represented by *Poikilolaimus*, which is formally classified within Rhabditidae [2,3]. This placement is consistently recovered in 18S rRNA, multigene and phylogenomic analyses [13,15,64] suggesting a more complex pattern of diversification within this group of nematodes than morphology suggests.

The Diplogasteromorpha sensu De Ley and Blaxter [2,3] is one of the branches stemming from the node R3 in Figure 5b and is monophyletic in all examined publications (excluding the family Odontopharyngidae; its taxonomic placement was discussed under Section 3.4), however the topology within this clade (R4 in Figure 5b) does not support superfamilies and most of the families it represents, except for the Cylindrocorporidae and monotypic Tylopharyngidae (not included in [2,3]), Mehdinematidae and Pseudodiplogasteroididae [65,66,67]. The remaining families Diplogasteroididae, Diplogastridae and Neodiplogasteridae are not monophyletic, while Cephalobiidae, Longibuccidae and Heteropleuronematidae (the latter two not included in [2,3]) have not been sequenced yet. Thus, the strategy proposed by Sudhaus and Fürst von Lieven [68] to treat this entire clade (R4 in Figure 5b) as a single taxon Diplogastridae, without subdivisions, became broadly accepted by majority of nematologists.

The families Mesorhabditidae and Peloderidae are recovered as sister taxa in a monophyletic Mesorhabditoidea (R6 in Figure 5b) not only in 18S rRNA based studies, but in the analysis involving several ribosomal and protein coding genes [64]. Sister to them (R5 in Figure 5b) is a clade that includes paraphyletic Rhabditidae (R7 in Figure 5b), which not only includes smaller monophyletic Diploscapteridae, Agfidae and Angiostomatidae [15,69,70,71] (the latter family was not listed or discussed in [2,3]), but also a monophyletic and strongly supported clade (R8 in Figure 5b) that includes animal parasitic Strongyloidea. Within Strongyloidea, Heterorhabditidae is a sister taxon to the remaining taxa (R8 in Figure 5b) consistently in single and multi-gene analyses [13,14,15]. The phylogenetic relationships within the remaining Strongyloidea (R9 in Figure 5b) are not clearly resolved in studies utilising rRNA loci [13,72], with all families (except for Diaphanocephalidae represented by a single sequence) being paraphyletic or polyphyletic depending on the used data. Thus, the consensus cladogram on the Figure 5 is based on the well-resolved phylogenomic analysis that included representatives of Trichostrongylidae, Heligmosomidae, Metastrongylidae, Ancylostomatidae and Strongylidae [15], and supplemented with 18S rRNA data for the families Molineidae and Diaphanocephalidae [13].

The position of the family Rhabdiasidae remains unresolved, despite availability of molecular data. Although De Ley and Blaxter [2,3] included this family in Panagrolaimomorpha, another opinion prevails that places it within Rhabditomorpha [73].

## 4. Discussion

A substantial percentage of the phylogenies examined and included in this study are based on the 18S rRNA region, with phylogenies based on other ribosomal loci, multigene datasets, genomes and transcriptomes included where possible. The dominance of 18S rRNA gene in phylogenetic analyses can be traced all the way back to when this gene was first utilised in what would be the first phylum-wide DNA-based analysis of nematode phylogeny [1]. The region has since had the reputation as being the most suitable marker for analysis of nematode phylogeny, especially when the scope spans the entire phylum. It was even regarded as sufficiently robust to deal with deeper phylogenies. Earlier phylogenies based on this gene, however, could not resolve the relationship at the root of Nematoda at the point of splitting between Enoplia, Dorylaimia and Chromadoria [12,16,17,18,21,24]. It was thought that higher taxon sampling might perhaps enhance the resolution of the relationships within Nematoda. However, as more taxa continued to be sampled into phylogenetic analysis, it became apparent that this region alone was incapable of resolving relationships between this enormous number of taxa [13]. This limited resolving power of the 18S rRNA gene was in part what drove the move towards the use of multiple gene loci or whole genome/transcriptome data for phylogenetic inference. Results from recent genome-based analyses have in fact demonstrated the improvement in the resolution of the nematode tree achievable through the use of whole genome and transcriptome datasets [14,15]. The most notable case points to the support for the sister relationship between Enoplia and the rest of Nematoda, previously only marginally supported by the 18S rRNA gene. Within the phylum, there are also groups such as Dorylaimida, Strongyloidea, Tylenchina, Spirurina and Cephalobomorpha for which the 18S rRNA gene outright is unfit as a marker for analysing phylogeny.

The most taxon-rich phylogenetic analysis based on the 18S rRNA gene to date amassed over 2700 sequences [13]. This follows the trend of increasing the number of taxa with each iteration of phylum-wide phylogenetic analysis based on the 18S rRNA [1,12,13,16,21]. With this increase in taxon sampling comes another challenge in having to exercise strict scrutiny of datasets included. The need to ensure that only high-quality sequences from accurately identified individuals are included in phylogenetic analyses cannot be overemphasised. The results of failure to adhere to this have been seen in analyses where sequences presumed to belong to the same taxon have occupied distant clades on the tree [7]. It is not just misidentified sequences that inadvertently find their way into analyses that always create this issue; sometimes these problematic sequences originate from correctly identified specimens (see placement of *Richtersia* spp. in [7]). In fact, such cases of “erroneous” sequences coming from properly identified samples (not mis-identified) are still an issue for single-locus-based phylogenies. However, these will eventually be “left behind” in phylogenomic analyses (and unfortunately replaced with misidentified or contaminated genomes and transcriptomes [14,15]). In barcoding and metabarcoding studies of nematodes where the 18S rRNA gene remains an important reference for OTU identification, these “erroneous” sequences will remain a big issue, unless they are specifically searched for and removed from reference databases. Even the intragenomic polymorphisms in 18S rRNA gene that have a negative impact on the phylogenetic analysis can be overcome in metabarcoding [74].

## 5. Conclusions

A better strategy for analysing nematode phylogeny involves targeting multiple genes instead of one. This approach is gaining more and more strength. With this, the sheer multitude of genes can help cancel out the ill effect of recalcitrant genes in the analysis. If available resources do not allow for sequencing whole genomes/transcriptomes or multiple genes, it is important to exercise caution when interpreting results of 18S rRNA-based phylogenies, especially in instances where these analyses suggest radical departure from previous hypothesis of relationships between species. Different taxa exhibit varying rates of genetic mutations, and this means it is unlikely that any single marker can perform equally well for inferring phylogeny across all groups of nematodes. A useful approach, if the only option is to use a single locus, may be to identify the most appropriate marker for a group of interest and utilise this region for analysis of phylogeny. For instance, by having a higher rate of mutation, the COI or the 28S rRNA regions perform better as phylogenetic markers than the 18S rRNA for some nematode lineages. In other words, for genus or family level analysis of phylogeny, single genes can still offer a wealth of information for reconstructing phylogeny. On the other hand, if the scope is an entire phylum, the improvement in resolution of relationships that whole genome/transcriptome or multigene sequences provide is worth the effort.

## Data Availability

Molecular data generated to support this study is available at NCBI GenBank under the following accession numbers: OL388444-OL388492 and OL671195.

## Supplementary Materials

The following are available online at https://www.mdpi.com/article/10.3390/ani11123479/s1, Figure S1: Phylogeny of Enoplia inferred by the PHASE3 Bayesian Inference; Figure S2: Phylogeny of Chromadoria inferred by the PHASE3 Bayesian Inference.
