# Supplementary figures and images for: Twenty Years after De Ley and Blaxter—How Far Did We Progress in Understanding the Phylogeny of the Phylum Nematoda?
Source: Animals (Basel). 2021 Dec 7;11(12):3479. doi: 10.3390/ani11123479 (PMC8697950; doi:10.3390/ani11123479)

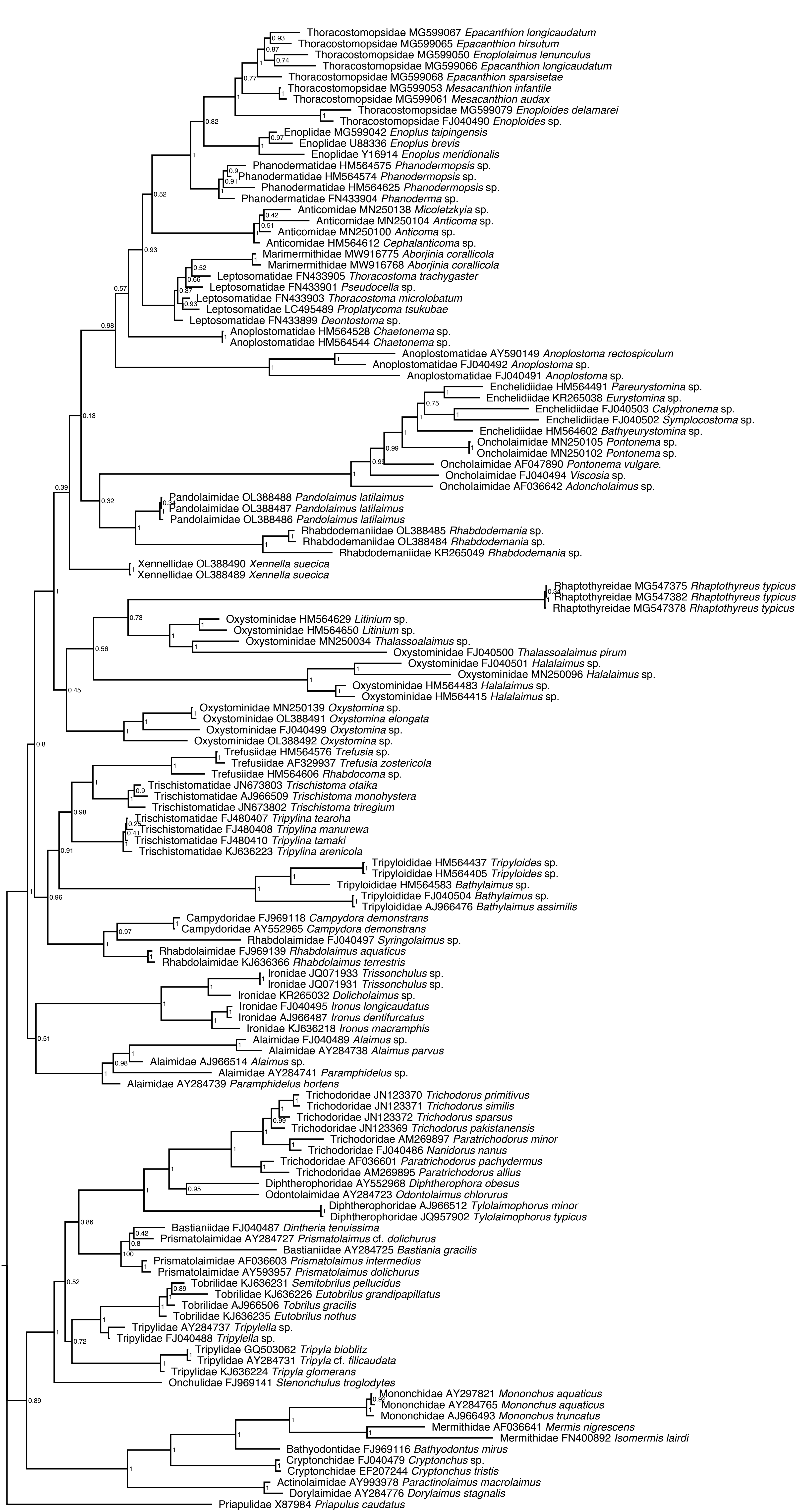

Supplement: Supplementary file 1 [file animals-11-03479-s001.zip › Figure S1.pdf]
