# Supplementary material for: Twenty Years after De Ley and Blaxter—How Far Did We Progress in Understanding the Phylogeny of the Phylum Nematoda?
Source: Animals (Basel). 2021 Dec 7;11(12):3479. doi: 10.3390/ani11123479 (PMC8697950; doi:10.3390/ani11123479)
Supplement: Supplementary file 1 [file Figure S2.pdf › Figure S2.pdf]

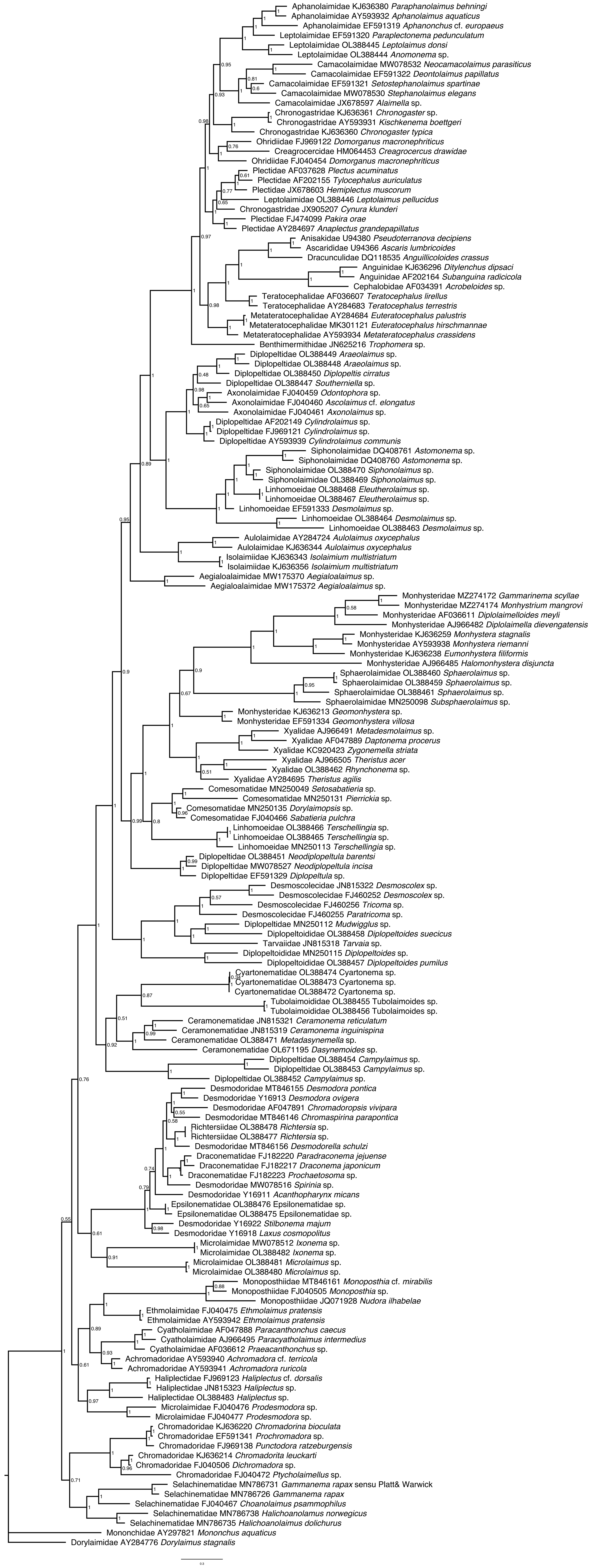
Aphanolaimidae KJ636380 *Paraphanolaimus behningi*
Aphanolaimidae AY593932 *Aphanolaimus aquaticus*
Aphanolaimidae EF591319 *Aphanonchus* cf. *europaeus*
Leptolaimidae EF591320 *Paraplectonema pedunculatum*
Leptolaimidae OL388445 *Leptolaimus donsi*
Leptolaimidae OL388444 *Anomonema* sp.
Camacolaimidae MW078532 *Neocamacolaimus parasiticus*
Camacolaimidae EF591322 *Deontolaimus papillatus*
Camacolaimidae EF591321 *Setostephanolaimus spartinae*
Camacolaimidae MW078530 *Stephanolaimus elegans*
Camacolaimidae JX678597 *Alaimella* sp.
Chronogastridae KJ636361 *Chronogaster* sp.
Chronogastridae AY593931 *Kischkenema boettgeri*
Chronogastridae KJ636360 *Chronogaster typica*
Ohridiidae FJ969122 *Domorganus macronephriticus*
Creagrocercidae HM064453 *Creagrocercus drawidae*
Ohridiidae FJ040454 *Domorganus macronephriticus*
Plectidae AF037628 *Plectus acuminatus*
Plectidae AF202155 *Tylocephalus auriculatus*
Plectidae JX678603 *Hemiplectus muscorum*
Leptolaimidae OL388446 *Leptolaimus pellucidus*
Chronogastridae JX905207 *Cynura klunderi*
Plectidae FJ474099 *Pakira orae*
Plectidae AY284697 *Anaplectus grandepapillatus*
Anisakidae U94380 *Pseudoterranova decipiens*
Ascarididae U94366 *Ascaris lumbricoides*
Dracunculidae DQ118535 *Anguillicoloides crassus*
Anguinidae KJ636296 *Ditylenchus dipsaci*
Anguinidae AF202164 *Subanguina radicicola*
Cephalobidae AF034391 *Acrobeloides* sp.
Teratocephalidae AF036607 *Teratocephalus lirellus*
Teratocephalidae AY284683 *Teratocephalus terrestris*
Metateratocephalidae AY284684 *Euteratocephalus palustris*
Metateratocephalidae MK301121 *Euteratocephalus hirschmannae*
Metateratocephalidae AY593934 *Metateratocephalus crassidens*
Benthimermithidae JN625216 *Trophomera* sp.
Diplopeltidae OL388449 *Araeolaimus* sp.
Diplopeltidae OL388448 *Araeolaimus* sp.
Diplopeltidae OL388450 *Diplopeltis cirratus*
Diplopeltidae OL388447 *Southerniella* sp.
Axonolaimidae FJ040459 *Odontophora* sp.
Axonolaimidae FJ040460 *Ascolaimus* cf. *elongatus*
Axonolaimidae FJ040461 *Axonolaimus* sp.
Diplopeltidae AF202149 *Cylindrolaimus* sp.
Diplopeltidae FJ969121 *Cylindrolaimus* sp.
Diplopeltidae AY593939 *Cylindrolaimus communis*
Siphonolaimidae DQ408761 *Astomonema* sp.
Siphonolaimidae DQ408760 *Astomonema* sp.
Siphonolaimidae OL388470 *Siphonolaimus* sp.
Siphonolaimidae OL388469 *Siphonolaimus* sp.
Linhomoeidae OL388468 *Eleutherolaimus* sp.
Linhomoeidae OL388467 *Eleutherolaimus* sp.
Linhomoeidae EF591333 *Desmolaimus* sp.
Linhomoeidae OL388464 *Desmolaimus* sp.
Linhomoeidae OL388463 *Desmolaimus* sp.
Aulolaimidae AY284724 *Aulolaimus oxycephalus*
Aulolaimidae KJ636344 *Aulolaimus oxycephalus*
Isolaimiidae KJ636343 *Isolaimium multistriatum*
Isolaimiidae KJ636356 *Isolaimium multistriatum*
Aegialoalaimidae MW175370 *Aegialoalaimus* sp.
Aegialoalaimidae MW175372 *Aegialoalaimus* sp.
Monhysteridae MZ274172 *Gammarinema scyllae*
Monhysteridae MZ274174 *Monhystrium mangrovi*
Monhysteridae AF036611 *Diplolaimelloides meyli*
Monhysteridae AJ966482 *Diplolaimella dievengatensis*
Monhysteridae KJ636259 *Monhystera stagnalis*
Monhysteridae AY593938 *Monhystera riemanni*
Monhysteridae KJ636238 *Eumonhystera filiformis*
Monhysteridae AJ966485 *Halomonhystera disjuncta*
Sphaerolaimidae OL388460 *Sphaerolaimus* sp.
Sphaerolaimidae OL388459 *Sphaerolaimus* sp.
Sphaerolaimidae OL388461 *Sphaerolaimus* sp.
Sphaerolaimidae MN250098 *Subsphaerolaimus* sp.
Monhysteridae KJ636213 *Geomonhystera* sp.
Monhysteridae EF591334 *Geomonhystera villosa*
Xyalidae AJ966491 *Metadesmolaimus* sp.
Xyalidae AF047889 *Daptonema procerus*
Xyalidae KC920423 *Zygonemella striata*
Xyalidae AJ966505 *Theristus acer*
Xyalidae OL388462 *Rhynchonema* sp.
Xyalidae AY284695 *Theristus agilis*
Comesomatidae MN250049 *Setosabatieria* sp.
Comesomatidae MN250131 *Pierrickia* sp.
Comesomatidae MN250135 *Dorylaimopsis* sp.
Comesomatidae FJ040466 *Sabatieria pulchra*
Linhomoeidae OL388466 *Terschellingia* sp.
Linhomoeidae OL388465 *Terschellingia* sp.
Linhomoeidae MN250113 *Terschellingia* sp.
Diplopeltidae OL388451 *Neodiplopeltula barentsi*
Diplopeltidae MW078527 *Neodiplopeltula incisa*
Diplopeltidae EF591329 *Diplopeltula* sp.
Desmoscolecidae JN815322 *Desmoscolex* sp.
Desmoscolecidae FJ460252 *Desmoscolex* sp.
Desmoscolecidae FJ460256 *Tricoma* sp.
Desmoscolecidae FJ460255 *Paratricoma* sp.
Diplopeltidae MN250112 *Mudwigglus* sp.
Diplopeltoididae OL388458 *Diplopeltoides suecicus*
Tarvaiidae JN815318 *Tarvaia* sp.
Diplopeltoididae MN250115 *Diplopeltoides* sp.
Diplopeltoididae OL388457 *Diplopeltoides pumilus*
Cyartonematidae OL388474 Cyartonema sp.
Cyartonematidae OL388473 Cyartonema sp.
Cyartonematidae OL388472 Cyartonema sp.
Tubolaimoididae OL388455 Tubolaimoides sp.
Tubolaimoididae OL388456 Tubolaimoides sp.
Ceramonematidae JN815321 *Ceramonema reticulatum*
Ceramonematidae JN815319 *Ceramonema inguinispina*
Ceramonematidae OL388471 *Metadasynemella* sp.
Ceramonematidae OL671195 *Dasynemoides* sp.
Diplopeltidae OL388454 *Campylaimus* sp.
Diplopeltidae OL388453 *Campylaimus* sp.
Diplopeltidae OL388452 *Campylaimus* sp.
Desmodoridae MT846155 *Desmodora pontica*
Desmodoridae Y16913 *Desmodora ovigera*
Desmodoridae AF047891 *Chromadoropsis vivipara*
Desmodoridae MT846146 *Chromaspirina parapontica*
Richtersiidae OL388478 *Richtersia* sp.
Richtersiidae OL388477 *Richtersia* sp.
Desmodoridae MT846156 *Desmodorella schulzi*
Draconematidae FJ182220 *Paradraconema jejuense*
Draconematidae FJ182217 *Draconema japonicum*
Draconematidae FJ182223 *Prochaetosoma* sp.
Desmodoridae MW078516 *Spirinia* sp.
Desmodoridae Y16911 *Acanthopharynx micans*
Epsilonematidae OL388476 Epsilonematidae sp.
Epsilonematidae OL388475 Epsilonematidae sp.
Desmodoridae Y16922 *Stilbonema majum*
Desmodoridae Y16918 *Laxus cosmopolitus*
Microlaimidae MW078512 *Ixonema* sp.
Microlaimidae OL388482 *Ixonema* sp.
Microlaimidae OL388481 *Microlaimus* sp.
Microlaimidae OL388480 *Microlaimus* sp.
Monoposthiidae MT846161 *Monoposthia* cf. *mirabilis*
Monoposthiidae FJ040505 *Monoposthia* sp.
Monoposthiidae JQ071928 *Nudora ilhabelae*
Ethmolaimidae FJ040475 *Ethmolaimus pratensis*
Ethmolaimidae AY593942 *Ethmolaimus pratensis*
Cyatholaimidae AF047888 *Paracanthonchus caecus*
Cyatholaimidae AJ966495 *Paracyatholaimus intermedius*
Cyatholaimidae AF036612 *Praeacanthonchus* sp.
Achromadoridae AY593940 *Achromadora* cf. *terricola*
Achromadoridae AY593941 *Achromadora ruricola*
Haliplectidae FJ969123 *Haliplectus* cf. *dorsalis*
Haliplectidae JN815323 *Haliplectus* sp.
Haliplectidae OL388483 *Haliplectus* sp.
Microlaimidae FJ040476 *Prodesmodora* sp.
Microlaimidae FJ040477 *Prodesmodora* sp.
Chromadoridae KJ636220 *Chromadorina bioculata*
Chromadoridae EF591341 *Prochromadora* sp.
Chromadoridae FJ969138 *Punctodora ratzeburgensis*
Chromadoridae KJ636214 *Chromadorita leuckarti*
Chromadoridae FJ040506 *Dichromadora* sp.
Chromadoridae FJ040472 *Ptycholaimellus* sp.
Selachinematidae MN786731 *Gammanema rapax* sensu Platt& Warwick
Selachinematidae MN786726 *Gammanema rapax*
Selachinematidae FJ040467 *Choanolaimus psammophilus*
Selachinematidae MN786738 *Halichoanolamus norwegicus*
Selachinematidae MN786735 *Halichoanolaimus dolichurus*
Mononchidae AY297821 *Mononchus aquaticus*
Dorylaimidae AY284776 *Dorylaimus stagnalis*
